# Association between overweight and characteristics of young adult students: support for nursing care^1^

**DOI:** 10.1590/0104-1169.0174.2549

**Published:** 2015

**Authors:** Jênifa Cavalcante dos Santos Santiago, Thereza Maria Magalhães Moreira, Raquel Sampaio Florêncio

**Affiliations:** 2 Doctoral student, Universidade Estadual do Ceará, Fortaleza, CE, Brazil. Professor, Universidade Estadual do Ceará, Fortaleza, CE, Brazil. Scholarship holder from Coordenação de Aperfeiçoamento de Pessoal de Nível Superior (CAPES), Brazil; 3 Post-doctoral fellow, Faculdade de Saúde Pública, Universidade de São Paulo, São Paulo, SP, Brazil. Professor, Universidade Estadual do Ceará, Fortaleza, CE, Brazil; 4 Master's student, Universidade Estadual do Ceará, Fortaleza, CE, Brazil. Scholarship holder from Coordenação de Aperfeiçoamento de Pessoal de Nível Superior (CAPES), Brazil

**Keywords:** Nursing, Overweight, Obesity, Young Adult

## Abstract

**OBJECTIVE:**

to verify associations between overweight and the characteristics of young adult students to support nursing care.

**METHOD:**

case-control study conducted with young adults from public schools. The sample was composed of 441 participants (147 cases and 294 controls, with and without excess weight, respectively). Sociodemographic and clinical characteristics were collected together with exposure factors and anthropometrics. Multiple logistic regression was used. The study received Institutional Review Board approval.

**RESULTS:**

statistically significant association with overweight: non-Caucasian, having a partner; weight gain during adolescence, mother's excess weight, the use of obesogenic medication, augmented diastolic blood pressure, of abdominal circumference and waist/hip ratio. In addition to these, schooling and weight gain during childhood were also included in the multivariate analysis. After adjustment, the final model included: having a partner, weight gain during adolescence, augmented diastolic blood pressure and abdominal circumference.

**CONCLUSION:**

the analysis of predictor variables for excess weight among young adult students supports nurses in planning and developing educational practices aimed to prevent this clinical condition, which is a risk factor for other chronic comorbidities, such as cardiovascular diseases.

## Introduction

Overweight and obesity are conditions on the rise among young adults and nurses need to work on the prevention of these conditions to improve the quality of life of this population.

One report conducted by the World Health Organization (WHO) states that obesity is the cause of death of 2.8 million people a year and 12% of the current world population is considered obese. A total of 26% of the adults on the American continent is obese; the region in which the condition is the most incident in the world. The WHO's department of statistics reports that obesity doubled between 1980 and 2008 around the world^(^ 1 ^)^.

In Brazil, excess weight is considered an important nutritional disorder. Accumulation of body fat often begins during childhood and adolescence and persists into adulthood, possibly leading to physiopathological effects in adulthood^(^ 2 ^)^.

The determination of overweight and obesity results from a set of factors that constitute the lifestyle of modern populations, consuming increasingly processed, energy-dense foods rich in sugar, fat and sodium, with calories above individual needs. This imbalance stems partly from changes in dietary patterns, combined with reduced exercise in both work and leisure activities^(^ 3 ^)^.

It is important to stress multiple and heterogeneous determinants (biological, historic, ecological, social, cultural and political factors) of overweight. Environmental and social causes are in the sphere in which individuals have little or no ability to interfere^(^ 4 ^)^.

It is apparent, in this context, that obesity is not a problem of developed countries only; it also affects a portion of less favored populations. Therefore, government authorities as well as nurses and other health care workers need to mobilize to establish priorities and strategies to promote health and control health conditions.

It is worth noting that the emphasis concerning this public health problem in the school context has been directed to specific groups: children and adolescents^(^ 5 ^-^ 7 ^)^. The young adult population (20 to 24 years old) still attending school, however, has not been included in research, which justifies this study.

The question guiding this study was: Is there any association between weight excess and the characteristics of young adult students? What factors associated with overweight are susceptible to nursing interventions?

Therefore, studies analyzing intervenient factors are needed to support educational actions nurses have to develop when providing care to overweight young adult students. The objective of this study was to verify the association between overweight and characteristics of young adult students to support nursing care.

This study's objective was to verify the association between overweight and characteristics of young adult students to support nursing care. 

## Method

This observational case-control study of a quantitative nature was conducted with young adult students from public schools, including both overweight and obese individuals (case group) and individuals not classified as obese or overweight (control groups). 

The study setting was the city of Maracanaú, located in the Metropolitan region of Fortaleza, Ceará, Brazil, with a population of 200,797 inhabitants^(^ 8 ^)^. It is an urban region with a large number of industries and has the second highest budget revenue of Ceará. 

The study's dependent variable was overweight/obesity. The independent variables were organized in hierarchical blocks to enable the multivariate analysis. The first block was composed of sociodemographic variables and the second block was composed of clinical and exposure variables.

We calculated samples of case-control studies and obtained a minimum of 172 for the control group and 86 people for the case group, considering a proportion of 2:1, which ensures statistic and operational efficiency and control selection bias. Because this paper is derived from a larger funded project, we decided to include in the sample the total of cases collected (n=147 students with body mass index (BMI) > 25 kg/m²) and two controls for each case (n=294 people with BMI > 18.5Kg/m² and < 25 kg/m²), totaling 441 people.

All the participants were recruited in public schools in the city and met the inclusion criteria (being regularly enrolled, aged between 20 and 24 years old with BMI equal to or higher than 18.5 Kg/m²). Age and occupation were considered to match the case and control groups.

Data were collected from November 2011 to April 2012. One questionnaire addressing sociodemographic and clinical data together with anthropometric measures was applied. The variables: race, weight gain in childhood and in adolescence, paternal and maternal weight gain, smoking and alcohol consumption were self-reported. The daily ingestion of sugary and fatty foods and poor ingestion of fruits and greens was considered to be an unbalanced diet. Less than 150 minutes of moderate activity per week was considered sedentariness in accordance to the International Physical Activity Questionnaire (IPAQ).

The anthropometric measures were taken in a standardized manner. The abdominal circumference was measured with an inextensible tape measure putting clothing away, locating the midpoint between the iliac crest and the last rib. Measures below 88 cm for women and 102 cm for men were considered normal. The hip circumference was verified at the level of the anterior trochanter border and its relationship with the waist was also verified. The waist/hip ratio was considered normal among women with waist/hip=0.85 and waist/hip=0.95 was considered normal for men^(^ 9 ^)^. Weight and height were determined using an anthropometric scale for adult individuals. The participants were standing with their arms close to the body, wearing the least weight in clothing possible without shoes. 

The body mass index was computed only to assign the study's participants to either case or control group but the variable was not considered in the statistical analysis since its frequency was pre-established. 

A database was created in the Statistical Package for the Social Sciences (SPSS) version 20.0, where variables were intersected and data were statistically analyzed.

Descriptive statistics (mean and standard deviation) were used and associations among risk factors were verified through the non-parametric Chi-square test between the explanatory variables and outcome, with level of significance at 5%, to perform the multivariate analysis. Afterwards, we verified the strength of the association among variables by using odds ratio and respective confidence intervals. Then, logistic regression analysis was performed to adjust for potential confounding effects considering p<0.20. The factors were hierarchically selected into blocs for the final model, considering p<0.05.

This study followed the ethical and legal precepts concerning research conducted with human subjects and received approved from the Institutional Review Board at the State University of Ceará (UECE), No. 11516679-3.

## Results

The description and analysis involved the predictor variables (sociodemographic, clinical-epidemiological, and exposure aspects) and outcome variables (overweight or obesity), as presented in Table 1.

**Table 1 -:** Bivariate analysis of sociodemographic characteristics associated with overweight/obesity in young adult students. Maracanaú, CE, Brazil, 2012

Variable	Cases BMI≥ 25 kg/m²			Controls 25 kg/m² >BMI ≥ 18.5Kg/m²			p*	Raw odds ratio (CI 95%)
	*f*	%	Mean (SD)	*f*	%	Mean (SD)		
Age								
20 to 22 years old	114	77.60%	21.9 (±1.42)	253	86.10%	20.93 (±1.19)		1
23 to 24 years old	33	22.40%		41	13.90%		0.325	1.79 (1.07-2.97)
Sex								
Male	71	48.30%		150	51.00%			1
Female	76	51.70%		144	49.00%		0.590	1.12 (0.75-1.66)
*Self-reported race*								
Caucasian	57	38.80%		79	26.90%			1
Non-Caucasian	90	61.20%		215	73.10%		0.011	0.58 (0.38-0.88)
Marital status								
Partner	50	34.00%		45	15.30%			1
No partner	97	66.00%		249	84.70%		0.000	2.85 (1.79-4.55)
*Family Income*								
Up to 2 times MW†	117	79.60%		237	80.60%			1
More than 2 times MW	30	20.40%		57	19.40%		0.800	1.07 (0.65-1.75)
*Live in Slums*								
Yes	11	7.50%		19	6.50%			1
No	136	92.50%		275	93.50%		0.689	1.17 (0.54-2.53)
Grade								
High School	90	62.10%		158	55.60%			1
Senior year (Grade 12)	55	37.90%		126	44.40%		0.202	0.77 (0.51-1.15)
*Father’s schooling*	**		**		**	**	**	
Up to 8 years	75	51.00%		147	50.00%		0.950	1
More than 8 years	39	26.50%		77	26.20%		0.804	1.07 (0.61-1.89)
Do not know	33	22.40%		70	23.80%		0.976	0.99 (0.62-1.60)
*Mother’s schooling*								
Up to 8 years	82	55.80%		155	52.70%		0.817	1
More than 8 years	36	24.50%		75	25.50%		0.849	1.06 (0.59-1.92)
Do not know	29	19.70%		64	21.80%		0.691	0.91 (0.56-1.47)

* Chi-square statistical significance;

† Minimum wage (MW) current in Brazil in 2012: R$ 622 Reais

As shown in Table 1, most participants from both groups (case and control) were aged between 20 and 22 years, were Non-Caucasians, had no partner, had a family income of up to two times the minimum wage, did not live in slums, were attending 1^st^ or 2^nd^ year of high school, and parents had attended up to eight years of school. Only the sex variable showed difference between groups: women were predominant in the case group while men predominated in the control group.

Table 2 presents the clinical characteristics of the young adult students verified by taking measures using appropriate equipment and technique. 

**Table 2 -:** Bivariate analysis of clinical and exposure characteristics associated with overweight/obesity in young adult students. Maracanaú, CE, Brazil, 2012

Variable	Cases BMI ≥ 25 kg/m²			Controls 25 kg/m² >BMI ≥18.5Kg/m²			p*	Raw Odds Ratio (CI 95%)
	*f*	%	Mean (SD)	*f*	%	Mean (SD)		
Weight during childhood								
No overweight	123	83.70%		265	90.10%		0.051	1
Overweight	24	16.30%		29	9.90%			1.78 (1.01-3.19)
Weight during childhood								
No overweight	108	73.50%		281	95.60%		0.000	1
Overweight	39	26.50%		13	4.40%			7.81 (4.01-15.19)
Father’s weight gain								
No overweight	120	81.60%		240	81.60%		1.000	1
Overweight	27	18.40%		54	18.40%			1.00 (0.60-1.67)
Father’s weight gain								
No overweight	97	66.00%		228	77.60%		0.010	1
Overweight	50	34.00%		66	22.40%			1.78 (1.15-2.76)
Systolic blood pressure								
Normotensive	142	96.60%	117.01 (±13.46)	285	96.90%	112.05 (±11.99)	0.848	1
Hypertension	5	3.40%		9	3.10%			1.12 (0.37-3.39)
Diastolic blood pressure								
Normotensive	127	86.40%	75.67 (±14.43)	288	98.00%	68.76 (±10.37)	0.000	1
Hypertensive	20	13.60%		6	2.00%			7.56 (2.97-19.27)
Abdominal circumference								
Normal	99	67.30%	M:91.85 (±9.86) F:90.27 (±10.11)	291	99.00%	M:76.51 (±5.50) F:74.74 (±6.66)	0.000	1
Altered ^†^	48	32.70%		3	1.00%			47.03 (14.33-154.36)
Waist/hip ratio								
Normal	107	72.80%	M:0.86 (±0.62) F:0.85 (±0.74)	260	88.40%	M:0.82 (±0.08) F:0.80 (±0.09)	0.000	1
Altered ^‡^	40	27.20%		34	11.60%			2.86 (1.72-4.76)
Smoking								
No	129	87.80%		250	85.00%		0.439	1
Yes	18	12.20%		44	15.00%			0.79 (0.44-1.43)
Alcohol								
No	41	29.90%		85	32.10%		0.660	1
Yes	96	70.10%		180	67.90%			1.11 (0.71-1.73)
Unbalanced diet								
No	71	48.30%		126	42.90%		0.279	1
Yes	76	51.70%		168	57.10%			0.80 (0.54-1.20)
Sedentariness								
No	28	19.00%		53	18.00%		0.794	1
Yes	119	81.00%		241	82.00%			0.93 (0.56-1.55)
Obesogenic medication								
No	104	70.70%		233	79.30%		0.048	1
Yes	43	29.30%		61	20.70%			1.58 (1.01-2.49)

* Chi-square test's level of significance

† > 88 cm for women and > 102 for men;

‡ > 0.85 for women and > 0.95 for men.

Predominance of the following variables was verified in both cases and controls: normal weight during childhood and adolescence, absence of weight excess among parents, normal systolic and diastolic blood pressure, and normal abdominal circumference and waist/hip ratio, no smoking, no alcohol consumption, no unbalanced diet, no sedentariness or obesogenic medications. 

Statistically significant associations were found in the bivariate analysis between the following sociodemographic characteristics and overweight/obesity (p<0.05): Being non-Caucasian and having a partner. For the following clinical and exposure characteristics, statistically significant associations were found for overweight and obesity (p<0.05): excess weight during adolescence; mother's history of excess weight; altered diastolic blood pressure; augmented abdominal circumference; augmented waist/hip ratio; and the use of obesogenic medication. Variables had to present association p<0.20 to be included in the adjustment of the logistic regression model. Hence, being a senior student (grade 12) and excess weight during childhood were also selected for the multivariate analysis.

After identifying variables p<0.20 we proceeded to the multivariate analysis with the adjustment stage (Table 3).

**Table 3 -:** Multivariate analysis of sociodemographic characteristics (block 1), clinical characteristics and other exposure factors (block 2), associated with overweight/obesity in young adult students. Maracanaú, CE, Brazil. 2012

Variables	p	Raw odds ratio	p*	Adjusted odds ratio (95%CI)
Block 1				
Self-reported race				
Caucasian		1.00		1.00
Non-Caucasian	0.0110	0.58 (0.38-0.88)	0.030	0.62 (0.40 – 0.96)
*Marital status*				
No partner		1.00		1.00
Partner	0.000	2.85 (1.79-4.55)	0.000	2.95 (1.82 – 4.78)
Schooling				
High School		1.00		1.00
Senior year (Grade 12)	0.202	0.77 (0.51-1.15)	0.549	0.88 (0.57 – 1.34)
Block 2				
Weight during childhood				
No overweight		1.00		1.00
Overweight	0.051	1.78 (1.01-3.19)	0.823	1.09 (0.52 – 2.26)
*Weight during adolescence*				
No overweight		1.00		1.00
Overweight	0.000	7.81 (4.01-15.19)	0.000	6.46 (2.98 – 13.99)
*Mother’s weight gain*				
No overweight		1.00		1.00
Overweight	0.010	1.78 (1.15-2.76)	0.374	1.28 (0.74 – 2.19)
*Diastolic blood pressure*				
Normotensive		1.00		1.00
Hypertensive	0.848	1.12 (0.37-3.39)	0.001	6.04 (2.10 – 17.38)
*Abdominal circumference*				
Normal		1.00		1.00
Altered	0.000	47.03 (14.33-154.36)	0.000	54.47 (13.55 – 219.04)
*Waist/hip ratio*				
Normal		1.00		1.00
Altered	0.000	2.86 (1.72-4.76)	0.274	0.62 (0.26 – 1.46)
*Unbalanced diet*				
No		1.00		1.00
Yes	0.279	0.80 (0.54-1.20)	0.085	0.66 (0.41-1.06)
*Obesogenic medication *				
No		1.00		1.00
Yes	0.048	1.58 (1.01-2.49)	0.429	1.26 (0.71 – 2.22)

* p: Chi-square level of significance

When analyzing the effect of sociodemographic characteristics (block 1) on overweight/obesity, being non-Caucasian and having a partner remained significant (p<0.05). The analysis concerning the effect of clinical characteristics and exposure (block 2) on overweight/obesity showed that excess weight during adolescence, hypertension (based on diastolic blood pressure) and augmented abdominal circumference were significant (p<0.05). Waist/hip ratio was not significant due to the strong correlation with the variable abdominal circumference (coefficient r de Pearson=0.502, p<0.001). This is expected since abdominal circumference is used to calculate the waist/hip ratio.

Table 4 presents the variables that remained in the final model. Multiple logistic regression was performed with the variables from blocks 1 and 2, which were p<0.05 in the adjustment stage. In this final stage, race did not remain significant and was withdrawn from the model, remaining only the marital status "have a partner", excess weight during adolescence, hypertension (diastolic arterial blood pressure) and augmented abdominal circumference.

**Table 4 -:** Final model of the logistic regression. Maracanaú, CE, Brazil, 2012

	B (standard error)	Confidence interval of 95% for Exp b		
		Inferior	Exp b	Superior
*Included*				
Constant	-6.59 (0.92) *			
*Marital status*	0.89 (0.29) †	1.40	2.44	4.27
Weight during adolescence	1.84 (0.38) *	2.98	6.30	13.31
Diastolic blood pressure	1.96 (0.54) *	2.49	7.11	20.28
Abdominal circumference	3.48 (0.62) *	9.59	32.42	109.54

Note: R²=0.20 (Hosmer and Lemeshow), 0.28 (Cox & Snell), 0.39 (Nagelkerke). X² model= 143.64, p<0.001.

* p<0.001,

† p<0.01.

Analysis of the residuals was performed to identify points in which the model had low adherence and points that improperly influenced the model. Standardized Cook's statistics were performed, considering DFBeta below 1 for all the variables and standardized residuals below 3, according to recommended parameters.

Table 4 shows that the final regression model was composed of constant, marital status, weight during adolescence, diastolic blood pressure, and abdominal circumference. All presented a positive relationship with the outcome, evidenced by Exp b > 1. Hence, the variables composing the final model are potential early indicators that lead to excess weight.

## Discussion

Having a partner, considered in this study as being in a stable union or married, was statistically associated with overweight/obesity in both the bivariate and multivariate analysis, and remained in the final model. One study^(^ 10 ^)^ conducted with the employees of a federal university verified that marital status was strongly associated with overweight and obesity, and prevalence was lower among single individuals, 41.8 and 1.8%, respectively. Another study^(^ 11 ^)^ found significant risk estimates (p<0.001) among men with a partner (PR=1.88). Another study^(^ 12 ^)^ conducted with adults in Maranhão verified that not having a partner was associated with lower prevalence of abdominal obesity (PR=0.28).

We suggest that marriage may influence weight gain due to changes in social behavior, which lead to increased caloric ingestion, due to foods rich in fat and sugar and decreased energetic expenditure, as a consequence of neglecting more rigorous physical activities and increased visits to restaurants and coffee shops as leisure. Additionally, couples tend to concern less with their self-image.

Current literature indicates that young adults are at risk of becoming obese or gaining excess weight when transitioning from childhood into adolescence or from adolescence into adulthood. The most critical periods of life for the development of obesity are early childhood, during the strong fluctuation in the trajectory of body fat that occurs between five and seven years old, and adolescence. From six years of age onwards, approximately one in every two obese children become an obese adult while only one in ten non-obese children become obese in adulthood^(^ 13 ^-^ 14 ^)^.

With regard to weight gain during adolescence, it was statistically significant in the bivariate analysis and remained in the final model when tested in the multivariate analysis, showing that young adults with a history of excess weight during adolescence are 6.3 times more likely to develop overweight/obesity in adulthood. 

Some studies report that obesity during childhood and adolescence is a concern because, if not controlled, the prognosis is increased morbidity and reduced life expectancy^(^ 15 ^-^ 16 ^)^, as it is associated with dyslipidemia, hypertension, glucose intolerance, psychosocial difficulties and increased risk of persistent obesity during adulthood^(^ 6 ^)^.

The literature usually addresses hypertension as a consequence of weight gain^(^ 7 ^,^ 17 ^)^. In this study, an association between hypertension as predictor variable and overweight/obesity as outcome could be identified. Both systolic and diastolic blood pressures were verified to check for hypertension and only the latter showed statistic significance as outcome, both in the bivariate and multivariate analysis, so that diastolic blood pressure remained in the final model. Hence, this study shows that hypertensive individuals are seven times more likely to develop overweight/obesity.

One cross-sectional study^(^ 18 ^)^ applied multiple logistic regression to verify the variables associated with overweight and obesity and the adjusted analysis showed that hypertension was one of the variables that were statistically associated (p<0.05) with overweight. Hypertensive individuals were 3.3 times more likely to develop overweight than normotensive ones. The analysis of obesity showed that hypertensive individuals were five times more likely to develop abdominal obesity that normotensive individuals.

One study^(^ 11 ^)^ verified in the bivariate analysis that men who self-reported hypertension presented a prevalence ratio 1.44 times higher of overweight and obesity; for women this ratio was 1.72. After applying Poisson regression, self-reported hypertension remained significant for both sexes. 

Abdominal circumference is used to classify different degrees of abdominal obesity, as well as cardiovascular risk, and is another indicator used in epidemiological studies, in addition to BMI. This accumulation of fat in the abdominal region is considered a risk factor for other diseases, such as endocrine, metabolic and cardiovascular diseases, even when BMI is within normal parameters^(^ 12 ^)^. Based on this fact, we investigated the association between this variable and the outcome, which was detected in the bivariate and multivariate analyses. The final model showed that abdominal circumference above normal parameters, which characterizes central obesity, increases 32 times the likelihood of a young adult to develop overweight or obesity. 

It is important to note that all the variables addressed in this study have some relationship with overweight/obesity. How strong this relationship is, however, depends on specific populations. The purpose of nurses is to identify which predisposing factors are interfering in the outcome through statistical analysis and then use their clinical and critical rationale to direct practices, be these practices to promote health or to prevent diseases or associate morbidities. 

The statistical analysis permits proposing some guiding points to support the clinical practice of nurses delivering care to overweight or non-overweight young adult students, directing the practice of health education. 

These points include considering that obesity involves biological, historical, ecological, economic, cultural, social and political factors, the causes of which are not only individual, but also environmental and social. Nurses should work to reach young adult students who do not usually seek the conventional health service network, and visit social spaces such as schools to promote the establishment of bonds between the health unit and the school, enabling the implementation of a quality multidisciplinary service to prevent diseases and promote health. 

It is worth noting that nurses are supposed to provide nursing consultations, monitor anthropometric data and ask for complementary exams to assess those at risk and refer these individuals to specialized professionals when necessary; identify together with the young adult what factors contributed, contribute or will contribute to overweight, from the perspective of diet, social and sportive behavior, and together seek strategies to overcome these factors; to overcome environmental and social factors, nurses and young adults should use the strategies proposed by public health policies and health care insurance/plans concerning the clinical condition; when overweight has already settled in, methods that minimize the risk of associated morbidities should also be used.

It is important to consider that nurses should have the support of other health care workers providing integral and interdisciplinary care to overweight or non-overweight young adults; all intervenient factors should be addressed by the nurse, considering the strategy to promote the health of young adult students who have not yet become overweight by using educational practices; and finally, nurses should always update their knowledge concerning research addressing the clinical care they provide, in addition to conduct studies to contribute to the implementation of such care.

## Conclusion

After bivariate analysis and adjustment of the multivariate analysis, the following variables remained in the final model: having a partner, overweight during adolescence, diastolic blood pressure indicating hypertension and augmented abdominal circumference.

All the variables tested in this study were subject to be statistically associated with overweight/obesity because they were based on various studies developed on this topic. One has to consider, however, the age interval of this young population addressed in this study. Many of the predictor variables would require longer periods to fully manifest. Other factors may have contributed to a lack of association: e.g. the accuracy of the answers provided by the participants was not take into account.

The conclusion, therefore, is that the analysis of the predictor variables for excess weight in young adult students support nurses in the planning and development of educational practices aimed to prevent this clinical condition, a risk factor for other chronic comorbidities, such as cardiovascular diseases.
